# Leveraging wearable haptics for guidance in virtual rehabilitation: effects on motor control from an immersive VR setting

**DOI:** 10.1038/s41598-026-35092-6

**Published:** 2026-02-04

**Authors:** Ali KhalilianMotamed Bonab, Cristian Camardella, Federica Serra, Antonio Frisoli, Federico Posteraro, Daniele Leonardis

**Affiliations:** 1 Institute of Mechanical Intelligence and the Department of Excellence in Robotics & AI, Scuola Superiore Sant’Anna, Pisa, Italy; 2 Azienda USL Toscana Nord Ovest (ATNO), Pisa, Italy

**Keywords:** Wearable haptics, Rehabilitation, Immersive, Virtual reality, Tactile, Neurorehabilitation, Biomedical engineering, Mechanical engineering, Rehabilitation

## Abstract

Immersive Virtual Reality (iVR) interventions have emerged as effective complements to conventional rehabilitation, with advantages in terms of flexible parametrization, extended data recording and patient engagement. Home-rehabilitation is another impactful, yet unexplored potential. A key requirement is ensuring that patients execute tasks with proper motor coordination and postural awareness: to this end, tactile sensory feedback can be introduced to enhance both immersion and motor guidance. We investigate here the efficacy and the effects on motor coordination of tactile guidance provided within an iVR rehabilitation serious game. A wearable haptic armband, delivering directional continuous and vibrotactile feedback, has been developed to guide grasping and pronosupination tasks. We evaluated the interaction on motor control and task execution using objective performance metrics, biomechanical responses, and subjective assessments, in a group of 12 healthy subjects, and a preliminary feasibility study with two participants with stroke. Findings show that haptic guidance in iVR statistically significantly enhances movement precision, reduces variability, and induces adaptive changes in muscle coordination. Feedback from clinical tests further indicates preliminary indications of usability and acceptability of the developed technology as part of a rehabilitation program. These results underscore the promise of haptic-enriched iVR systems for advancing both clinical and home-based motor rehabilitation.

## Introduction

The neurorehabilitation following neurological impairments, such as stroke, neurodegenerative diseases, or chronic neuromotor disorders, often necessitates intensive, supervised therapy to restore motor functions^1,2^ and improve daily life activities^3,4^. While conventional rehabilitation methods in clinical settings are proven to be effective in motor recovery of patients^1,2^, most patients are required to undergo a prolonged rehabilitation process^5–7^. Such a need for extended rehabilitation treatments is constrained by the limited resources of healthcare systems, involving access to dedicated infrastructures and supervision by trained clinical personnel. Also, patients’ adherence to the treatment is challenged by motivation and possible difficulties in reaching these specialized rehabilitation centers ^8^.

Novel neurorehabilitation methods have been developed to complement traditional approaches, showing also the potential to address the above challenges, for example, enabling patients to continue therapy outside clinical settings with minimal direct physician supervision^9^. Among these methods, virtual reality (VR) rehabilitation has emerged as a widely experimented therapeutic modality^10,11^, providing intriguing capabilities with respect to conventional therapy. They encompass fine and flexible parametrization of the exercise, as well as repeatability of the intervention and extensive data recording, allowing for a better estimation of the patient’s progress and more robust scientific comparisons^12,13^. In addition, a key element of VR rehabilitation is the serious-games approach: it proposes rehabilitation exercises in the shape of virtual games, with the purpose of fostering engagement of the patients and, in turn, their motivation and active mental participation in the therapy^14–16^.

Building on the success of VR-based rehabilitation, immersive virtual reality (iVR) represents a significant leap forward in therapeutic interventions, and it has been used as a tool to engage patients in virtual activities and therapeutic exercises specifically designed to promote their neurological recovery^17^. By providing enriched virtual environments that support multilevel sensory interactions, iVR further enhances motor learning and functional recovery^11^. Consequently, several studies have investigated the efficacy of iVR-based rehabilitation methods, such as mirror therapy and motor imagery, in different populations, including stroke survivors^18–21^, patients with multiple sclerosis^22^, individuals experiencing phantom limb pain^23,24^, and children with cerebral palsy^15,25^.

While VR-based rehabilitation allows patients to repeat training sessions and practice in a controlled virtual environment, it is critical to ensure that patients perform tasks with proper kinematics and motor coordination comparable to that of healthy individuals^11^. This consideration becomes especially important in envisaging home-based rehabilitation, where the absence of direct clinical supervision increases the risk of deviating from the desired movement patterns. Given the human body kinematics and muscular redundancy, the development of non-optimal compensatory movement strategies ^26,27^ can arise in the unsupervised virtual rehabilitation setting.

It emerges the need for guidance in motor execution, which, similarly to the physical intervention of the therapist to correct body movements, can be conveyed through the tactile sensory pathway. In virtual reality settings, motor guidance is typically implemented via visual feedback that aims to promote correct motor learning in rehabilitation. These systems often utilize graphical elements such as bars, directional arrows, and target trajectories, as well as phantoms or virtual trainers that demonstrate the desired movement patterns^28–30^. The primary goal of such guidance is to provide real-time, intuitive cues that help users adjust their motions during task execution, thereby reinforcing correct movement strategies. This often involves comparing the user’s ongoing performance to predefined reference trajectories and visualizing deviations from the expected path. In some cases, targets or movement goals are displayed within the virtual environment to direct the user’s attention and facilitate precise motor execution. Considering scaling to a wider number of degrees of freedom and body segments in postural guidance, it appears convenient to avoid the cognitive overload of the visual channel, and the need of a line of sight with the represented visual cues. The introduction of tactile feedback in VR has been largely explored to provide touch sensation in otherwise visual-only interactions^31^. To this purpose, informative tactile information can be provided by a variety of compact wearable devices^32^. Being designed to stimulate mechano-receptors only, and not to provide motor assistance, these interfaces show advantages in compactness, cost, usability and safety for unsupervised applications with respect to other robotic interfaces experimented in clinical rehabilitation^33^. Similar systems have already been proposed in immersive VR rehabilitation to increase engagement and multi-sensory congruence of the performed motor task^15,34^. Specifically in the context of motor guidance, haptic feedback is often used to provide clear directional cues through complex devices such as exoskeletons, physically guiding the user’s limbs along desired trajectories^35^. Additionally, it can serve to alert users when their movements stray from predefined targets or deviate from the intended path^36^.

The tactile sensory input has the potential to not only increase immersion but also to serve as an active guide for motor control^11^. The haptic cues can reinforce proper movement patterns and reduce the risk of compensatory strategies, increasing the effectiveness of neurorehabilitation exercises.

Although the integration of wearable haptic feedback into virtual and immersive virtual reality is well established, its role in rehabilitation as motor guidance remains underexplored ^37,38^. It emerges broader evidence of wearable devices experimented in navigation tasks, conveying directional information through haptic bracelets^39–41^. Outside the rehabilitation and VR area, haptic feedback has been proposed for postural correction^42,43^, investigating how tactile cues can guide trunk balance and improve posture in lifting tasks.

**Fig. 1:**
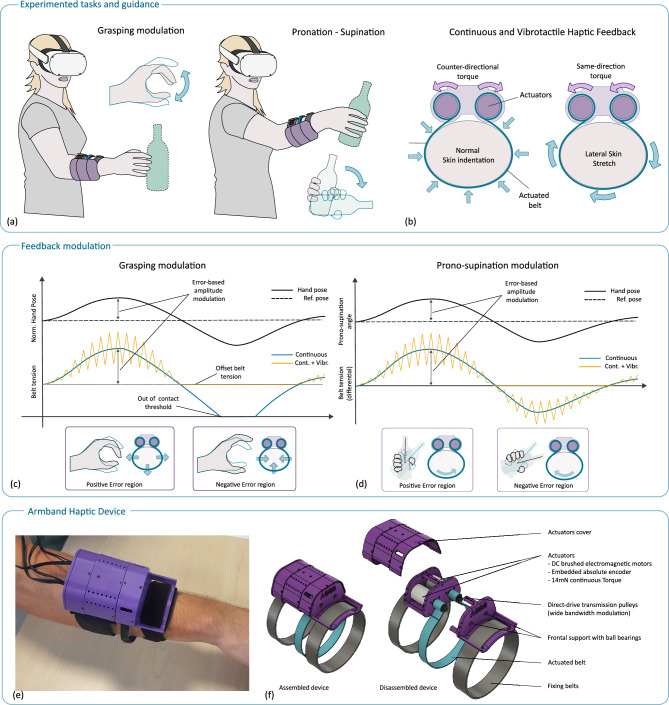
(**a**) Illustration of the pick-and-place task with pronosupination and two movement phases guided by haptic feedback. (**b**) The operation principle of the wearable haptic device delivering both clenching (normal skin indentation) and lateral stretch stimuli in a wide modulation bandwidth. (**c**) Haptic modulation strategy: feedback intensity is adjusted based on the amplitude of the measured pose error (current hand grasp or pronation angle, represented by the solid gray hand) relative to a reference (wireframe hand). Two modalities were tested: continuous modulation and continuous plus vibrotactile feedback. In grasping, feedback primarily signals excessive hand closure, while hand opening is limited by the offset tension and the point when the belt loses contact. (**d**) For pronosupination, the lateral stretch feedback is modulated symmetrically around the zero-error point, with the direction of stretch matching the sign of the angular error; differential belt tension on the y-axis reflects the force disparity between the two motors. (**e**) Photograph of the developed armband prototype used in the study. (**f**) Computer-Aided Design (CAD) representation of the device and its internal components, highlighting the direct-drive mechanism that delivers wide bandwidth continuous and vibrotactile stimuli.

Motivated by the above, this study provides a systematic investigation of the effects of tactile feedback motor guidance in immersive VR rehabilitation. We apply here tactile feedback for postural guidance of the upper limb within an immersive VR rehabilitation exercise (Fig. 1). The haptic guidance targets in particular pronosupination and opening-closing of the hand, two functions included in the conventional pick-and-place rehabilitation exercise^44,45^. We systematically evaluate the effects in a group of healthy participants executing precise motor control, analyzing their biomechanics and kinematics to determine whether haptic feedback influences movement patterns, potentially leading to adaptations in motor execution or changes in muscle coordination, in response to the added reference-following task. We also investigate the effectiveness of haptic guidance, comparing two different approaches in rendering modulated haptic cues, based on continuous and vibrotactile signals. To assess user experience, we evaluate perception and workload. Finally, we conducted a feasibility evaluation of the approach with two post-stroke patients, exploring their perception of the device, workload, and acceptance of the technology as a potential tool for neurorehabilitation.

## Results

**Fig. 2:**
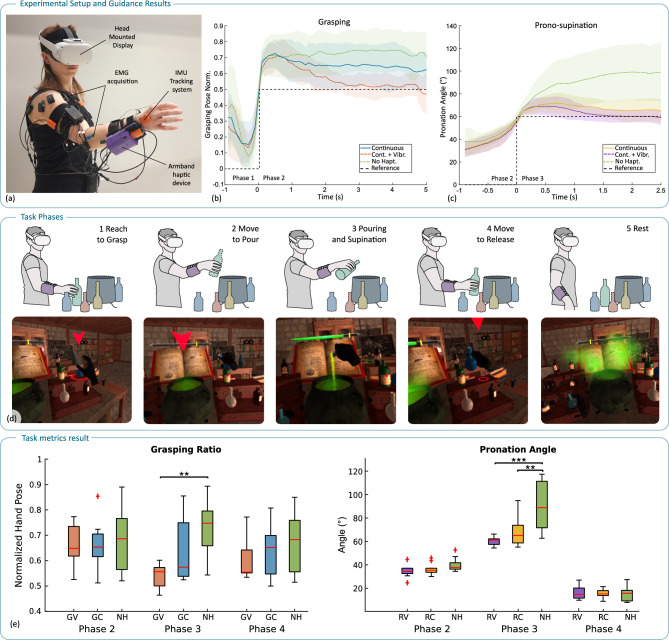
(**a**) The experimental setup, including monitoring of the upper limb kinematics and muscular activation with sEMG, in addition to the vision-based tracking embedded in the 3D headset. (**b**) Averaged transient signals of the hand grasping ratio at the beginning of the grasping phase (time 0): it depicts modulation of the hand grasping in response to the different delivered feedback stimuli. Starting from phase 1, the feedback is delivered in the GC and GV conditions in order to guide the subject toward the reference hand pose (dotted line). (**c**) transient signals of the measured pronation angle, here at the beginning of the pouring phase (time 0). The guiding effect of the provided feedback is visible from the beginning of phase 3. (**d**) Task sequence presented to participants in immersive virtual reality, based on the serious game developed for rehabilitation of the upper limb. The participant is asked to prepare a magic potion through the following phases: (1) The player grabs the selected (red arrow) ingredient using full-hand grasping. (2) The ingredient is moved over the cauldron in preparation for pouring. (3) When the ingredient is rotated over an angular threshold, it starts pouring into the cauldron. (4) Pouring is stopped by rotating the ingredient back above the angular threshold, and then the original position on the shelf is reached. 5) When the ingredient is released, a 5-second stop interval is introduced before starting a new repetition. (**e**) Aggregated results of the metrics directly related to the grasping modulation and pronosupination task.

We evaluated the effects of haptic guidance in a pick-and-place exercise with pronosupination in a group of nine healthy subjects. Due to either data loss or calibration issues, the total sample size of 12 subjects was used for the questionnaire analysis only. Then, we explored the feasibility of the approach in a clinical setting, with an experimental rehabilitation session involving two participants with stroke. In the virtual environment, both the grasping and the pronosupination actions were setpoint-based motor tasks, simulating a clinical scenario in which setpoints are parametrized as a progressive goal of the motor exercise. Our analysis was threefold: first, we measured objective performance metrics, assessing effectiveness of the haptic guidance; second, we examined the interaction of haptic guidance with motor control and execution using biomechanical data; finally, we collected subjective measures of workload and user perception of the haptic feedback on both healthy subjects and individuals with stroke. These evaluations were conducted across five conditions, investigating guidance on two motor actions (grasping and pronosupination) and in two different feedback modalities, continuous (C) and combined continuous plus vibrotactile feedback (V) (Fig. 1). The five conditions were: grasping continuous (GC), grasping continuous + vibrotactile feedback (GV), no haptic feedback (NH), rolling continuous (RC), and rolling continuous + vibrotactile feedback (RV) (Fig. 2). The NH condition provided no guidance to the participant. In reporting the results below, mean and standard deviation values have been reported for each metric and condition, plus the 95% confidence interval (CI).

With the experimental setup depicted in Fig. 2a and throughout each exercise phase Fig. 2c, we monitored two key metrics: the hand grasping ratio, normalized from 0 (fully open) to 1 (fully closed), and the hand pronosupination angle (positive angles toward pronation). Transients depicted in Fig. 2b,c clearly show the reaction of the subject to the onset of the feedback at the beginning of the grasping phase (phase 1 to phase 2) and of the pronosupination phase (phase 2 to phase 3). In all the feedback conditions, after an initial overshoot, subjects adjusted the hand pose closer to the reference. The continuous vibrotactile feedback appears to be the most effective as regards precision, with the pose error reduced below 10% in $2.5\,s$ and $1.2\,s$ for the grasping and pronosupination modulation, respectively. Notably, the high within-subject variability observed under the NH condition (green area in Fig. 2b,c) further underscores the importance of haptic motor guidance in achieving optimal performance: without guidance, subjects accomplish the goal using different strategies from one trial to another, exceeding the setpoints of the virtual exercise, and leading to a higher variability. When receiving the haptic guidance, not only does the motor action get closer to the reference, but also the variability decreases, indicating the guiding feedback could be clearly perceived and followed. Results aggregated for each phase and condition, depicted in Fig. 2e, confirm the transient results. Starting from phase 2 and throughout phase 3 and phase 4, participants modulated the grasping ratio effectively in particular with the V feedback, achieving hand poses that were closer to the desired reference and with noticeably smaller variance between subjects;

The V feedback resulted significantly closer to the reference in phase 3 (phase 2 – GV: $0.66~\pm ~0.08$, CI = $[0.60,\ 0.72]$; GC: $0.66~\pm ~0.10$, CI = $[0.58,\ 0.74]$; NF: $0.69~\pm ~0.13$, CI = $[0.58,\ 0.80]$; phase 3 – GV: $0.53~\pm ~0.07$, CI = $[0.48,\ 0.58]$; GC: $0.63~\pm ~0.12$, CI = $[0.53,\ 0.73]$; NF: $0.73~\pm ~0.12$, CI = $[0.63,\ 0.83]$, $p = 0.002$ for GV vs NF ($d = -2.71$); phase 4 – GV: $0.59~\pm ~0.08$, CI = $[0.53,\ 0.65]$; GC: $0.64~\pm ~0.11$, CI = $[0.55,\ 0.73]$; NF: $0.67~\pm ~0.12$, CI = $[0.57,\ 0.77]$).

Haptic feedback played a crucial role in guiding participants to achieve the precise pouring angle associated with proper pronosupination in phases 2 and 3. As shown in Fig. 2e, the mean pouring angle was significantly closer to the reference in the RV condition and in the RC condition, compared to NH (phase 2 – RV: $36.25~\pm ~6.13$, CI = $[31.50,\ 41.00]$; RC: $37.34~\pm ~4.89$, CI = $[33.38,\ 41.30]$; NF: $40.80~\pm ~5.80$, CI = $[36.14,\ 45.46]$; phase 3 – RV: $61.60~\pm ~3.70$, CI = $[58.76,\ 64.44]$; RC: $69.24~\pm ~11.74$, CI = $[60.22,\ 78.26]$; NF: $91.82~\pm ~20.58$, CI = $[76.42,\ 107.22]$, $p = 0.007$ for RC vs NF ($d = -2.25$) and $p = 0.0003$ for RV vs NF ($d = -1.79$); phase 4 – RV: $17.59~\pm ~5.57$, CI = $[13.32,\ 21.86]$; RC: $17.06~\pm ~3.37$, CI = $[14.17,\ 19.95]$; NF: $16.94~\pm ~5.89$, CI = $[12.52,\ 21.36]$).

Overall, the combination of continuous and vibrotactile (V) feedback yielded superior performance than (C), with participants achieving more accurate and consistent hand poses than with continuous feedback alone.

### Grasping biomechanics

**Fig. 3:**
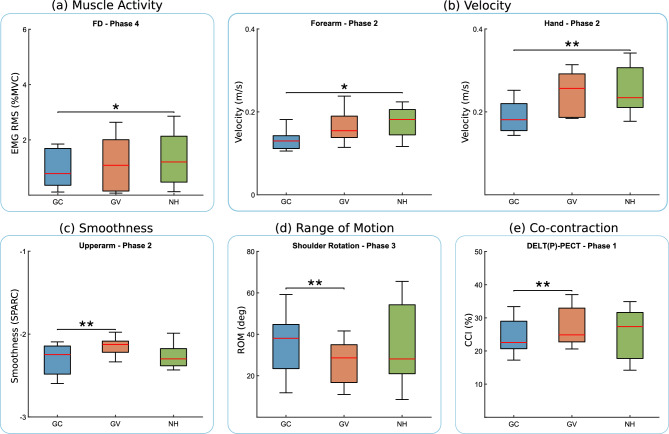
Comparison of muscle activation (**a**), segment velocity (**b**), smoothness (**c**), range of motion (**d**), and co-contraction (**e**) across participants in grasping (GC, GV) and baseline (NH) conditions. Brackets between conditions indicate significant differences. For the co-contraction panel (**f**), abbreviations denote specific muscles: deltoid (anterior, posterior) as DELT (A, P), and pectoralis as PECT. *Note* * indicates $p \le 0.017$; ** indicates $p \le 0.003$; and *** indicates $p \le 0.0003$.

**Fig. 4:**
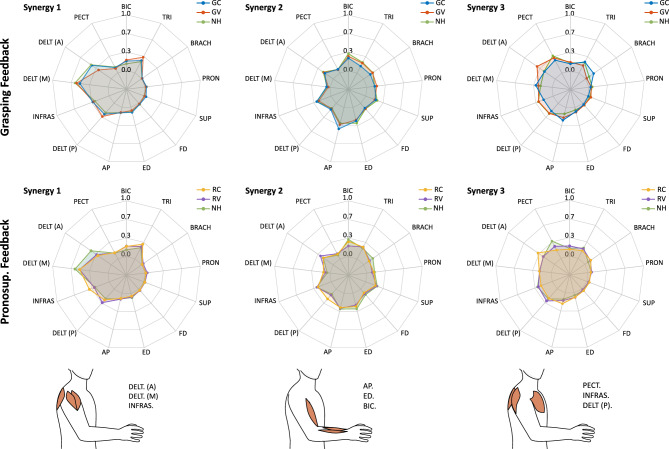
Radar plots depicting the mean muscle synergy weights for grasping (GC, GV) and pronosupination (RC, RV) conditions relative to the baseline (NH). Abbreviations denote specific muscles: BIC (biceps brachii), TRI (triceps brachii), BRACH (brachioradialis), PRON (pronator teres), SUP (supinator), FD (flexor digitorum), ED (extensor digitorum), AP (abductor pollicis), DELT (A, M, P) (deltoid (anterior, middle and posterior)), INFRAS (infraspinatus), and PECT (pectoralis major).

Analysis of biomechanical parameters indicates that participants adjusted their kinematics and muscle coordination in response to haptic feedback on grasping modulation. Regarding the movement kinematics (Fig. 3d), the range of motion (ROM) in grasping conditions (GV and GC) remained largely consistent with the NH condition across all phases, except in phase 3, where shoulder rotation ROM in GV ($21.62 \pm {3.50}$ deg, CI = $[19.29,\ 23.95]$) compared to GC ($23.19 \pm {13.24}$ deg, CI = $[13.96,\ 32.42]$) was significantly lower ($p = 0.003$, $d = -0.17$). Regarding movement motion-level kinematics (Fig. 3b, c), forearm ($0.13 \pm {0.02}$ m/s, CI = $[0.11,\ 0.14]$) and hand velocity ($0.19 \pm {0.04}$ m/s, CI = $[0.16,\ 0.21]$) in GC was significantly lower than NH (forearm: $0.17 \pm {0.04}$ m/s, CI = $[0.14,\ 0.20]$; hand: $0.25 \pm {0.06}$ m/s, CI = $[0.20,\ 0.29]$) during phase 2 (forearm: $p = 0.011$, $d = -1.20$; hand: $p = 0.003$, $d = -1.19$). Additionally, motion smoothness analysis showed that during the second phase of the game, the GC ($-2.32 \pm {0.19}$, CI = $[-2.47,\ -2.17]$) condition produced, compared to GV ($-2.16 \pm {0.12}$, CI = $[-2.25,\ -2.07]$), significantly smoother upper arm movements ($p = 0.007$, $d = -0.99$).

In terms of muscle coordination, the normalized RMS EMG signals (Fig. 3a) remained consistent across GC and GV during the first three phases. However, during the move to release phase (phase 4), subjects in the GC condition ($0.009 \pm {0.007}$, CI = $[0.0046,\ 0.0134]$) exhibited significantly lower flexor digitorum (FD) activity ($p = 0.007$, $d = -0.47$) compared to the NH condition ($0.013 \pm {0.010}$, CI = $[0.0063,\ 0.0197]$). Analysis of the co-contraction index (CCI) (Fig. 3e) further revealed that, in phase 1, the pectoralis-deltoid (posterior) muscle pair exhibited lower co-contraction ($p = 0.003$, $d = -0.60$) in the GC condition ($24.11 \pm {5.26}$, CI = $[20.05,\ 28.17]$) compared to GV ($27.47 \pm {6.05}$, CI = $[22.88,\ 32.06]$).

Finally, a comparison of muscle synergies between grasping conditions (GC and GV) and the NH baseline revealed a strong correlation (Fig. 4), indicating that participants did not substantially alter their synergies when haptic feedback was applied to grasping tasks. This suggests that haptic feedback facilitated motor execution without disrupting the natural coordination patterns of muscle activation.

### Pronosupination biomechanics

**Fig. 5:**
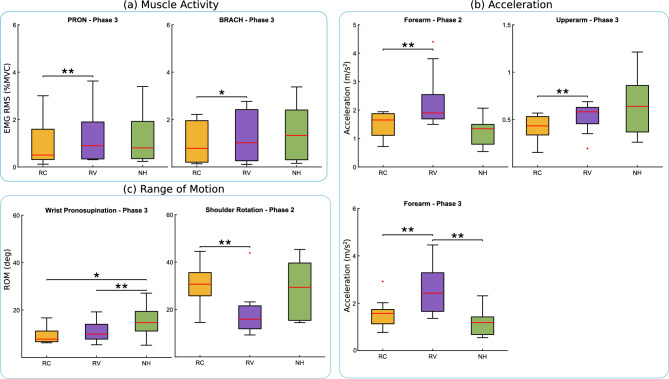
Comparison of muscle activation (**a**), segment acceleration (**b**), and range of motion (**c**) across participants in pronosupination (RC, RV) and baseline (NH) conditions. Brackets between conditions indicate significant differences. *Note* * indicates $p \le 0.017$; ** indicates $p \le 0.003$; and *** indicates $p \le 0.0003$.

The haptic feedback on pronosupination (i.e., RC and RV conditions) had a greater impact on the biomechanics and kinematics of the participants, as they needed to coordinate their entire arm to follow the reference, compared to the grasping modulation task, which only required modulation of the hand and fingers.

Regarding movement kinematics (Fig. 5c), participants exhibited a significantly lower range of motion (ROM) in shoulder rotation ($p = 0.003$, $d = -1.19$) during the moving and pronation phase (phase 2) when receiving multimodal feedback (RV: $18.63 \pm {10.65}$, CI = $[11.43,\ 25.83]$) compared to continuous feedback alone (RC: $30.60 \pm {9.51}$, CI = $[24.08,\ 37.12]$). In phase 3 (pouring and supination), the ROM in wrist pronosupination was significantly lower ($p = 0.011$, $d = -1.19$) in the RC condition ($9.38 \pm {3.64}$, CI = $[6.55,\ 12.21]$) compared to the no-haptic (NH) baseline ($15.68 \pm {6.56}$, CI = $[10.85,\ 20.51]$), with the RV condition also showing a significant reduction in wrist pronosupination ROM ($10.84 \pm {4.30}$, CI = $[7.63,\ 14.05]$) relative to NH ($p = 0.007$, $d = -0.87$).

Regarding motion-level kinematics (Fig. 3b), arm segment velocities remained relatively consistent with no significant differences observed in any phase of the game. However, arm acceleration showed more noticeable variation. In phase 2, forearm acceleration was significantly higher in the RV condition ($2.31 \pm {1.05}$, CI = $[1.56,\ 3.06]$) compared to the RC ($1.51 \pm {0.46}$, CI = $[1.16,\ 1.86]$) condition ($p = 0.003$, $d = 0.99$). Phase 3 exhibited greater variability, with forearm acceleration (RC: $1.56 \pm {0.65}$, CI = $[1.10,\ 2.02]$; RV: $2.65 \pm {1.10}$, CI = $[1.79,\ 3.51]$; NH: $1.16 \pm {0.55}$, CI = $[0.75,\ 1.57]$) significantly higher in both RC ($p = 0.007$, $d = 0.66$) and RV ($p = 0.007$, $d = 1.72$) conditions compared to NH. Furthermore, during this phase, the acceleration of the upper arm was significantly lower in RC ($0.42 \pm {0.13}$, CI = $[0.32,\ 0.52]$) condition compared to RV ($0.52 \pm {0.16}$, CI = $[0.40,\ 0.64]$) condition ($p = 0.007$, $d = -0.69$). Despite these variations, there were no significant changes in the smoothness of movement, though the increased variation in acceleration did lead to higher movement smoothness variability.

In terms of muscle biomechanics (Fig. 3a), overall muscular activity (RMS EMG) remained consistent during the first two phases of the game across conditions. However, in phase 3, the pronator teres ($p = 0.007$, $d = -0.18$) and brachioradialis ($p = 0.011$, $d = -0.42$) exhibited lower activation in the RC (PRON: $0.011 \pm {0.010}$, CI = $[0.0043,\ 0.0177]$; BRACH: $0.009 \pm {0.008}$, CI = $[0.0035,\ 0.0145]$) condition compared to RV (PRON: $0.013 \pm {0.012}$, CI = $[0.0057,\ 0.0203]$; BRACH: $0.013 \pm {0.011}$, CI = $[0.0061,\ 0.0199]$).

The co-contraction index showed no significant differences between the haptic conditions and the NH baseline. Notably, though, muscle synergy analysis (Fig. 4) revealed that participants altered their synergy patterns during the task execution with the RC condition compared to NH, as evidenced by a lack of significant correlation in the third synergy between RC and baseline conditions ($med(p) = 0.389$, $med(R_{pearson}) = 0.126$).

### User perception

**Fig. 6:**
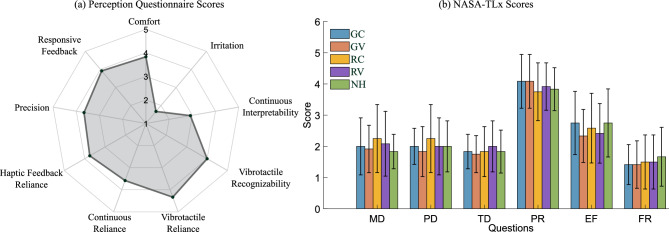
(**a**) Results from the user perception questionnaire, evaluating comfort, interpretability, and reliance on the feedback. (**b**) NASA-TLX perceived workload scores across conditions, showing mental, physical, and temporal demands, as well as effort and frustration.

To assess the psychological impact of each feedback method, participants completed the NASA-TLx after each condition and a custom perception questionnaire at the end of the session. Statistical analysis revealed no significant differences in perceived workload across conditions (Fig. 6b), indicating that the additional sensory stimuli from the haptic feedback did not alter the users’ subjective task load.

According to the perception questionnaire (rated on a 5-point Likert scale from 1 = strongly disagree to 5 = strongly agree), participants rated the device as comfortable (3.83 ± 0.98) and reported minimal irritation from the applied vibration and pressure (1.66 ± 0.84). They found the continuous feedback moderately interpretable (2.91 ± 0.95) and reliable (3.58 ± 1.18), while the multi-modal feedback (combining continuous and vibrotactile cues) was rated highly in terms of recognizability (4.00 ± 0.70) and reliance (4.33 ± 0.62). Overall, participants reported that they strongly depended on the haptic feedback to complete the tasks (3.75 ± 0.82), which helped guide their movements more precisely (3.66 ± 0.47), with the feedback being perceived as timely and responsive (4.39 ± 0.64). The results of the perception questionnaire are summarized in Fig. 6a.

**Fig. 7:**
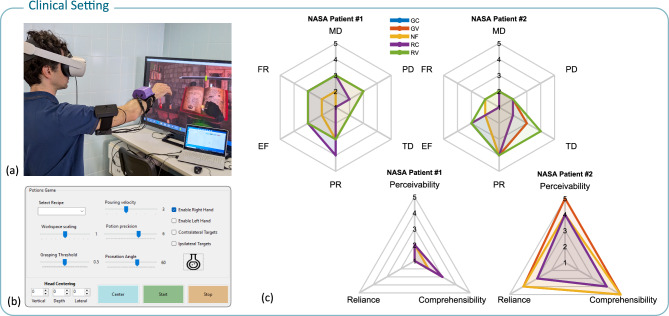
(**a**) Representative picture of the experimental clinical setting. Here, the setup is composed of the functional elements only: the VR headset with embedded tracking and the armband device. A wide monitor and a desktop PC were used to provide the VR visual streaming to the therapist and a control GUI with the parameters of the exercise. (**b**) Parameters of the serious game are tunable by the therapist, including the reference hand grasping ratio and the reference angle of pronation. Additional parameters are related to workspace dimensions, position of the target objects, dynamics and required precision of the pouring phase. (**c**) Questionnaire results of the experimental tests with two individuals with stroke, experiencing the immersive rehabilitation scenario and the haptic guidance in the different feedback conditions.

### Clinical feasibility

To evaluate the clinical feasibility of our iVR game with integrated haptic feedback, we conducted a single-session pilot study with two participants with stroke. Unlike the healthy participant trials, these sessions were performed with a minimal acclimation phase, and no quantitative biomechanical data were recorded to limit the setup time. After each condition, participants completed the NASA-TLx questionnaire along with three additional questions related to haptic feedback perception. At the end of the session, an interview was conducted to gather further general impressions.

The NASA-TLx results revealed that the first participant experienced moderate mental demand when receiving haptic feedback, with multimodal conditions (GV and RV) requiring more effort than continuous-only conditions. This participant also reported low temporal demand across all conditions and perceived high performance under continuous feedback, while overall effort and frustration were rated as moderate. In contrast, the second participant experienced low physical and mental demand, although the temporal demand was rated as high in the RV condition and moderate in the GV and GC conditions. This participant consistently rated performance as high, noted moderate effort (with the GV condition being the least effortful), and reported low frustration in all conditions.

Subjective perceptions further underscored these differences. The first participant indicated a low reliance on the haptic feedback for task completion, with moderate comprehensibility for multimodal feedback and lower comprehensibility for continuous-only feedback, leading to an overall low perceptibility rating. In contrast, the second participant rated reliability as high for both GV and RC conditions and moderate for GC and RV, while also finding GV and RC highly comprehensible, with the GV condition achieving the highest perceptibility score.

During the interviews, both participants with stroke expressed a willingness to incorporate the device into their rehabilitation programs, rating their likelihood as 4 out of 5 on a scale where 5 indicated strong agreement. Moreover, both strongly agreed (rating of 5) that the device could help improve their motor skills in a rehabilitation setting. Regarding comfort, the first participant with a stroke found the device to be highly comfortable, while the second rated it as moderately comfortable; neither participant experienced any irritation from the applied stimuli.

## Discussion

The role of haptic feedback in guiding the user within the proposed motor tasks has been evidenced by results related to performance metrics. In particular, transient signals and aggregated results in Fig. 2 evidence how, after the onset of the feedback, subjects could be guided toward the reference hand pose, in all the tactile feedback conditions, both for the grasping and pronosupination tasks. These two actions only had setpoint thresholds in the virtual exercise, which are intended to be set by the therapist as motor goals for patients. In this context, the reduction of the motor execution variability means a clear guidance could be provided, promoting the use of the approach for guiding specific and more precise motor strategies by the patient.

The V feedback resulted in the most effective, with higher precision of the subjects in following the reference task execution. The C feedback guidance appeared less effective, showing also greater performance variability among subjects, especially for the grasping task. Overall, these results are in agreement with those of the perception questionnaire, where participants rated the multimodal feedback (GV, RV) as highly interpretable and reliable, whereas the continuous feedback (GC, RC) was perceived as moderately reliable. Regarding interpretability, the mapping of the haptic feedback to the given motor tasks was designed to be as intuitive as possible, with the vibrotactile aiming at enhancing the perception of the continuous modulation. On the other hand, the number of actuators and the two modalities (continuous and vibrotactile) offer additional combinations that can possibly increase the informative degrees of freedom. Also, further investigations on the amplitude balance between the continuous and vibrotactile components can lead to optimization of the device design in terms of dimensions and output bandwidth.

Regarding the biomechanical analysis, we observed distinct differences in movement dynamics between the grasping conditions. Under the GC condition, participants tended to perform at lower speeds. In contrast, the GV (vibrotactile feedback) condition elicited a more impulsive movement strategy, with reduced smoothness in the upper arm segment. This discrepancy may reflect differences in how participants relied on the two feedback types: based on the perception survey, participants rated the vibrotactile feedback (GV) as highly interpretable and reliable, a finding that correlated with a more confident movement strategy. This approach necessitated fine motor adjustments and resulted in increased muscular engagement compared to the continuous cutaneous feedback condition. Specifically, the increased demand for precision in the GV condition was accompanied by significantly higher co-contraction in the anterior deltoid-pectoralis pairs. Such co-contraction is critical for shoulder joint stability, as it increases joint stiffness and limits unwanted motion, thereby providing a stable base for controlled, precise movements^46,47^. The observed reduction in flexor digitorum (FD) activity during the release phase may be attributed to its involvement in both wrist and finger flexion^48^. Despite this change in muscle activation, the overall muscle synergy remained stable across both GV and GC conditions, indicating that the fundamental coordination strategy was preserved.

During the RV condition, the range of motion (ROM) for shoulder rotation was significantly reduced compared to RC during the second phase. Similarly, the wrist pronosupination ROM during the third phase was significantly lower in both RC and RV compared to the NH condition. In general, the decreased inter-subject variability in ROM while receiving haptic feedback supports the device’s potential to provide clear hints during motor tasks.

Regarding movement dynamics, arm velocity did not differ significantly between the RC and RV conditions. However, forearm acceleration was consistently higher in the RV condition compared to RC, particularly during the targeted modulation phases. Additionally, due to the intermittent nature of vibrotactile feedback in the RV condition, subjects were prompted to react more quickly, resulting in higher acceleration responses to adjust to the provided cues. In contrast, the continuous cutaneous feedback in the RC condition offered a more sustained sensory input, leading to smoother, less accelerated arm movements.

In terms of muscle activation, during the supination phase, we observed significantly lower activity in the pronator teres and brachioradialis under the RC condition compared to RV. This finding suggests that the continuous cutaneous feedback in the RC condition provides a stable and consistent sensory cue, enabling participants to achieve the desired movement with less compensatory muscle activation, whereas the vibrotactile cue in the RV condition requires additional muscular effort to stabilize and adjust the forearm position (especially on brachioradialis when the arm is pronated)^49^.

Our analysis of muscle synergies revealed that under continuous rolling haptic feedback condition, the third synergy shifted from being pectoralis-dominant in the NH condition to one with a higher contribution from the anterior deltoid, accompanied by reduced contributions from the triceps and infraspinatus and an increased reliance on the brachioradialis. We interpret this reorganization as an adaptive response to the enriched sensory environment provided by the haptic feedback. Specifically, the anterior deltoid is known to play a critical role in stabilizing and positioning the arm when the shoulder is highly abducted^50^, making it well-suited for fine motor adjustments when additional tactile cues are available. Meanwhile, the increased contribution of the brachioradialis compared to the NH condition suggests that participants rely more on this muscle for forearm stabilization during precision tasks^49^.

The pilot evaluation with participants with stroke explored the feasibility of the approach in the clinical setting. Both individuals were able to perform an experimental rehabilitation session with the proposed methods, with parameters of the virtual exercises set by the therapist through the provided graphical interface (Fig. 7). Acceptability of the system by the two participants resulted in different attitudes, showing one participant more engaged by the novel approach, and the other less convinced, although still favorable for the possible introduction of the methods in his rehabilitation treatment. Both participants were able to accomplish the proposed iVR task with haptic feedback and reported overall low physical load and low-to-moderate cognitive load. Regarding the potential for home rehabilitation, envisaged as the long-term objective of the presented research, it appears on one hand promising as it concerns the overall setup and operability of the system. The developed components, together with modern off-the-shelf VR gear, suggest a potentially robust setup and simple calibration procedures. The introduced guidance is also a key element for the effective self-operability of such rehabilitation systems, increasing patients’ awareness of body posture and task execution. On the other hand, it is worth noting that operation without the physical presence of the therapist appears feasible only in a patient population showing a certain level of residual motor functionalities, since tactile interfaces cannot provide active motor assistance.

In literature, there are similar examples of approaches relying on tactile stimulation during motor exercises with two main goals: to provide a multi-sensory experience during rehabilitation, and to stimulate the sensory-motor channels, and/or guidance towards more correct motor patterns. With respect to alternative approaches using visual cues only (e.g.,^28–30^), haptic guidance offers the advantage of distributing information across modalities, in line with multiple resource theory, which predicts reduced interference when cognitive demands are spread across different sensory channels^51^. Evidence from multisensory integration supports this rationale: combining tactile and visual signals can improve performance under high perceptual load conditions^52^; Wang et al. showed that haptic cues allowed users to maintain visual attention on the primary task while still responding to guidance signals^53^; and Camponogara et al. demonstrated that haptic position cues improved performance when visual information was uncertain^54^. This distinction is particularly relevant in clinical populations, where stroke survivors frequently present with visual and attentional deficits, further limiting the capacity of the visual channel^55,56^. In such cases, complementing visual information with task-aligned haptic cues may provide a valuable means to support motor performance while mitigating attentional overload, and the approach can be scaled to additional degrees of freedom and body segments. There are also other applications of haptic feedback in clinical contexts, as in the work of Held et al.^57^ in which they tested a vibrotactile system to promote the use of the impaired side of upper limbs, against the learned non-use habit. Regarding multi-sensory stimulation, although broadly discussed in neurophysiology works that also include haptic devices, it lacks extensive and deep investigations^58^: very few cases achieved clinically relevant results, when compared to a control group, about the efficacy of long-term tactile stimulation^59^.

The guiding application can rely either on natural or metaphorical feedback, given by haptic devices or robots. Rehabilitation robots, exoskeletons in particular, thanks to their compliance with human biomechanics, are better suited to provide assistance and guidance during motor tasks at different body district levels, at the expense of much higher costs and the need for support by physical therapists. These systems rely on natural interactions, simulating direct support of therapists during exercises, providing torque at the articulations when needed (e.g., assist as needed control paradigm)^35^. On the other hand, haptic devices, in particular wearable devices, can be a good tradeoff between efficacy in guiding patients during motor tasks and the overall complexity and cost of systems. Most of these systems rely on metaphorical feedback, for example, vibrotactile stimulations, to guide patients during exercises, with a consequent higher cognitive load needed to contextualize the feedback to the motor guidance hint. Except for bulkiness and comfort, which are important elements to consider during the design, the intuitiveness of metaphors is crucial to achieving effective guidance. The proposed device showed the highest accuracy when continuous tactile feedback was combined with task-aligned vibrotactile modulation, where vibration intensity directly encoded performance error. This form of vibrotactile stimulation differs from metaphorical approaches, as it provides cues that are congruent with the ongoing motor task and require minimal interpretation. A similar outcome was reported by Devigne et al., who compared a wearable vibrotactile guidance system with a no-feedback condition in the context of power wheelchair navigation. Their system resulted in a significant reduction in collisions and improved spatial awareness^60^. These results highlight the effectiveness of modulated vibrotactile feedback as an intuitive and efficient cue for guiding motor tasks with minimal supervision. Similarly, Aggravi et al.^61^ developed wearable vibrotactile bracelets to guide visually impaired skiers by providing real-time directional cues through vibrations on the arms. Their system enabled non-visual and intuitive communication of movement instructions, enhancing safety and accuracy during skiing. Experimental results demonstrated that the task-aligned vibrotactile feedback reduced cognitive load compared to traditional vocal commands and improved users’ autonomy and confidence when navigating dynamic environments.

None of these works analyzed the effect of haptic feedback on either muscle synergies or range of motion metrics. In literature, many other devices have been evaluated from the task performance point of view, nevertheless, without considering users’ comfort or device usability. Liu et al. proposed a haptic system to evaluate wrist motor functions (in terms of RoM and task performance), designing the haptic feedback to warn the user when deviating from the correct trajectory rather than guiding them towards a setpoint^36^. Bark et al. tested a haptic armband that provides motor guidance through vibrotactile feedback on the forearm, emulating the gentle touch of physical therapists during correction actions^62^. These systems were revealed to be suitable for minimally supervised rehabilitation in cases of the presence of residual motor capabilities of patients. While the effectiveness of haptic feedback for motor learning has been established^63^, the present study focused specifically on its role in guiding precise upper-limb movements in an immersive rehabilitation context. Our results provide preliminary evidence that task-aligned haptic cues can improve movement precision and influence muscle coordination patterns during controlled exercises. Still, more structured investigations are needed to better understand the efficacy of haptic guidance in supporting precise motor execution within rehabilitation protocols, and to establish clinically relevant differences compared to control conditions. The efficacy of therapies should also be evaluated considering subjective assessment of comfort and usability, for a deeper understanding of potential mixed interaction effects between the intuitiveness of the guidance and the acceptability of the device. Moreover, the role of the therapist appears still pivotal in determining parameters for the exercise, as well as supervision and motivation of the patient beyond the specific motor functionalities. Integration in similar systems of telepresence technologies for clinical personnel seems a viable solution to introduce flexible, high-level remote supervision.

### Study limitations

This study has the following limitations that should be considered when interpreting the results. First, due to the complexity of the experimental setup, the sample size was limited to twelve healthy participants. On the clinical side, the system was evaluated with only two participants with stroke, which restricts generalization of the findings in rehabilitation contexts. Moreover, the clinical evaluation was limited to subjective feedback, as no kinematic or performance data were recorded from these participants. In addition, user perception data were collected using a custom, non-validated questionnaire, which may limit comparability with studies employing standardized instruments. The haptic guidance approach was also not compared against alternative modalities, such as visual or auditory cues, which constrains conclusions about its relative effectiveness. The study was conducted in a single-session, controlled laboratory setting and focused on two upper-limb movements, grasping and pronosupination. As such, long-term effects, generalization to broader motor tasks, and usability in unsupervised home-based environments remain open questions for future research.

## Conclusion

The study shows that multimodal haptic feedback has a relevant potential to enhance motor performance in typical rehabilitative upper-limb motor tasks, performed in immersive VR. Specifically, the combination of continuous and vibrotactile cues improved grasping and pronosupination precision, reducing their variability, and inducing adaptive changes in muscle coordination. This was evidenced by stable synergy patterns in most of the conditions, altered co-contraction, and modified muscle activation. Additionally, the preliminary feasibility evaluation with two participants with stroke provided initial indications of the system’s technical usability in a clinical setting, comfort, low physical and mental workload, and positive user impressions. These findings underscore the potential of haptic-enriched iVR systems to advance clinical rehabilitation, also paving the way to home-based rehabilitation, where appropriate supervision on the execution of the proposed exercises is one of the elements to be addressed.

## Methods

### Virtual reality serious game

The virtual environment is designed as a serious game scenario for the rehabilitation of the upper limb. It presents parametrized pick-and-place tasks with pronosupination, immersing the user in the role of a wizard apprentice. The player is proposed to prepare magical potions by picking and pouring the appropriate elements into a cauldron; if the task is correctly and precisely performed, a final reward is provided (a rabbit, present in the scene, is transformed into another animal). Parametrization of the game is one of the key advantages of rehabilitation based on virtual serious games. A graphical user interface is available for the therapist to adjust the main parameters of the game to the patient’s needs. It is possible to adjust the workspace dimension, select different potion recipes composed of easier or more difficult ingredients (placed on contra-lateral or higher shelves), set the reference hand pose required to grasp the ingredient, and set the pronation angle for pouring ingredients into the cauldron. The required precision and velocity of the pouring phase can also be tuned to adjust the difficulty and dynamics of the game. The game sequence is depicted and described in Fig. 2. The VR application has been developed in Unity®(version 2022.3.5f1, Unity Technologies, USA), and the implemented VR headset was an Oculus Quest 2 by Meta®(Meta Platforms Inc., USA). The vision-based hand-tracking system used in the headset is embedded in the device. A recent study by Abdlkarim et al.^64^ provided an evaluation of the tracking accuracy, reporting an average error in fingertip position of 1.1 cm and an average error in finger joint angle of $9.6^\circ$. In order to allow direct data comparison, the parameters of the game have been held fixed for all the healthy participants and feedback conditions.

### Haptic stimuli

Haptic feedback was rendered according to two primary actuation modalities: normal indentation and lateral stretch. First, normal indentation or clenching is produced when the two actuators rotate in opposite directions, causing the belt to tighten or loosen around the arm (Fig. 1b). This modality has been associated with the sensation of hand grasping and releasing, with increased pressure corresponding to a grasp and decreased pressure to a release. Lateral skin stretch is achieved when both motors rotate in the same direction, causing the belt to move laterally. An impedance control based on a position closed-loop was implemented for each motor in order to render the continuous clenching and lateral stretch actions, while the vibration component was driven in feed-forward. The lateral stretch modality has been related to guidance in the pronation and supination, ideally mimicking an external hand (i.e., the therapist) guiding the movement of the patient.

The above modalities can be modulated as continuous feedback with slow dynamics (as in^65^) or, through the novel device used in this study, as vibrotactile feedback. The direct-drive actuation allows the use of the same motors to propagate high-frequency vibrations through the actuated belt, modulating intensity and frequency independently. The effect of modulated vibrotactile feedback superimposed on continuous feedback was of particular interest to emphasize perception and, therefore, has been investigated in the study.

1 $$\begin{aligned} F_{a,b} = A_{a,b}e_p(K_c e_b + K_{v}sin(2\ pi f_v t)) \end{aligned}$$

Equation (1) represents the generic control law relating the linear pulling force $F_m$ applied by the motor A or B to the belt, depending on the measured pose error $e_p$. The pose error $e_p$ is related to the normalized pose of the hand (0 equal to fully open, 1 to fully closed) for the grasping task, and to the pronation angle in degrees for the pronosupination task. The error $e_b$ represents the displacement error of the belt used in the low-level impedance control, and is measured as a position error in mm by the actuator encoder. Depending on the feedback and task conditions, different values, listed below, were used for the coefficient weighting the pose error $A_{a,b}$, the coefficient $K_c$ (measured in N/mm) modulating the stiffness of the low-level impedance control for the continuous belt displacement, and the coefficient $K_v$ (measured in N) modulating the intensity of the vibrotactile feedback. The intensity and frequency of the vibrotactile feedback were set through a pilot experiment, with the aim of providing a clearly perceivable, yet comfortable stimulus, also at the maximum measured error.

According to the experimental conditions, the haptic stimuli were provided as follows:

- GC - when the subject’s hand pose was closer than the reference (positive error), a continuous clenching action, with belt displacement proportional to the pose error, was rendered from phase 2 to phase 4. Negative error corresponded to proportionally lower clenching displacement, up to the limit of full loosening of the belt around the arm. ($A_a=1$, $A_b=-A_a$, $K_c=0.006$, $K_v=0$).
- GV - analogous to GC, plus vibration added in the positive error region with intensity proportional to the pose error ($A_a=1$, $A_b=-A_a$, $K_c=0.6$, $K_v=1.8$, $f_{v} = 60$ Hz).
- RC - A continuous lateral stretch displacement, proportional to the pronation error, was rendered from phase 2 to phase 3. Direction of the lateral stretch was according to the pose error direction ($A_a = A_b = 0.05\,\, 1/deg$, $K_c=0.6$, $K_v=0$).
- RV - Analogous to RC, plus vibration added on the pulling actuator proportional to the pronation error ($A_a=A_b=0.05\,\, 1/deg$, $K_c=0.6$, $K_v=1.8$, $f_{v} = 60$ Hz).
- NH - No haptic feedback provided.

The maximum force applied to the belt by each actuator due to saturation of the nominal current intensity was $F_{max, belt}$ = 3.68 *N*.

### Haptic armband device

**Fig. 8:**
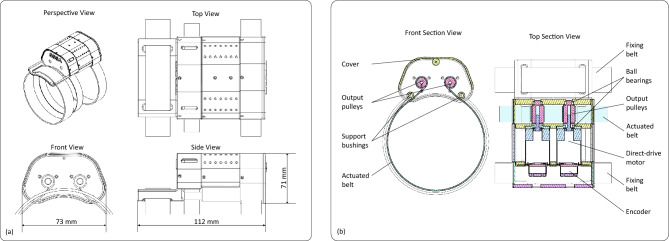
Design detail of the mechanical implementation of the wearable actuated unit (**a**) perspective and orthogonal views of the device; (**b**) details of the functional mechanical components in the frontal and top section views.

The implemented wearable haptic device is composed of five main components: the structural frame, an actuation unit, an actuated belt, control electronics, and the battery. The actuation unit comprises two direct-drive DC motors (DCX22S, Maxon Motor AG, Switzerland) with integrated absolute encoders (ENX 16 EASY Absolute, Maxon Motor AG, Switzerland). Dimensions of the device and details of the internal placement of components are depicted in Fig. 8. The output shaft is attached via a low-radius pulley to a flexible belt worn around the user’s arm. The mass of the device is 170 g for the module worn on the forearm with actuators, while it is 110 g for the module worn on the arm, which contains the battery and control electronics. Control electronics consists of an ESP32 microcontroller board (Adafruit ESP32 Feather, Adafruit Industries LLC, USA) and a custom board, integrating two H-bridge motor drivers (DRV8833C, Texas Instruments Inc., USA) for motor control and a DC-DC booster converter module (Pololu Corporation, USA) to regulate the power supply to the encoders. The device operates in wireless mode through WiFi communication and UDP protocol. The on-board control loop, driving the actuators, was computed at 1 ms sample time, while the rate of the haptic reference messages, received from the VR application, was 100 Hz.

The device is inspired by the work from Barontini et al.^65^, with the important difference that here the design implements direct-drive actuation, without using gear reducers. While this solution reduces the maximum output force, it allows wide-bandwidth actuation of the belt (158 Hz cutoff frequency, linear response from 0 to $\sim$20 Hz). The adopted design reduces non-linearities, friction losses, and significant reflected inertia typically introduced by geared transmissions. This simplicity at the joint level enables the implementation of an open-loop impedance control architecture for independent joint control, as the mid-level controller of the device. In this control scheme, the system uses joint position and velocity as inputs to compute the desired torque, and it does not rely on direct measurement of the output torque for feedback.

**Fig. 9:**
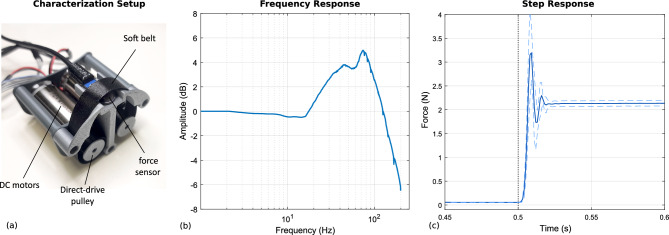
Characterization of the actuators and of the soft belt coupled with a force sensor (**a**). Frequency response to a chirp input signal (**b**). Step response (**c**): continuous line represents the mean of ten repetitions, dotted lines represent the standard deviation.

Characterization at the bench of the frequency and step response of the device has been conducted using an Optoforce 10N force sensor (2 mN resolution), coupled to the actuated belt of the device through a plastic support (Fig. 9a). A logaritmic chirp signal with 1.85 V amplitude and spanning from 0 Hz to 250 Hz in 30 s was used as the input signal. The frequency response (Fig. 9b) shows a flat behavior in the range of the continuous modulation driven by user movements (below 10 Hz). Then, it shows a resonant behavior with a peak at 74 Hz and a cutoff (-3 dB) at 158 Hz, due to the visco-elastic effect introduced by the soft belt. The step response was measured using a 1.85 V input step signal, repeated ten times. Results (Fig. 9c) reflect a similar behavior of the frequency response, showing an oscillatory transient at 81 Hz. The response is also highly repeatable, considering the limited standard deviation (dotted lines) measured during the rising edge transient. Numerical results of the step response are reported hereafter: rise time: 0.004 s, transient time: 0.0173 s, overshoot: 49.4%, peak: 3.19 N, steady state: 2.14 N, steady state error: 0.23 N (the error is computed with respect to the ideal actuator’s output force).

### Experimental procedure

#### Participants

Experiments were conducted with 12 healthy participants (7 male and 5 female; age: 29.4 ± 3.02 years, height: 172.4 ± 8.58 cm, weight: 68.08 ± 11.62 kg; mean ± standard deviation), in addition, a preliminary feasibility study of the system was performed with two male participants with stroke. All healthy participants were right-handed and self-reported no neurological, chronic, or physical impairments. One post-stroke individual (40 years old), classified as being in the chronic stage with a Fugl-Meyer Assessment (FMA) score of 39, exhibited neurological impairment in the left arm, while the other individual (68 years old), also in chronic with an FMA score of 44, presented with neurological impairment in the right hand. The experimental protocol was reviewed and approved by the Scuola Superiore Sant’Anna Review Board (approval number 41-2023). Both sets of participants were briefed on the procedures and provided their written informed consent before the experiments, which were conducted in accordance with the Declaration of Helsinki. The individuals depicted in Figs. 2 and 7 have provided explicit and written consent for the publication of their images.

Data measured from all the healthy subjects was used to compute metrics depicted in Fig. 2, while one subject was excluded from the biomechanical analysis due to calibration issues in the recorded data.

### Experimental protocol

The experimental protocol required participants to complete the iVR-based game under five conditions, all conducted on the same day in random order. These conditions, based on the various types of feedback provided by the haptic armband, include: grasping continuous (GC), grasping vibrotactile (GV), rolling continuous (RC), rolling vibrotactile (RV), and a baseline condition with no haptic feedback (NH). During all tasks, participants could always see their virtual hand pose in real-time, allowing them to adjust their movements accordingly. For the grasping phase, the system included a designated threshold for detecting a successful grasp (hand closure). Similarly, during the pronosupination phase, the liquid was poured only when the designated pronation angle threshold was reached. A visual progress bar displayed how much potion had been poured during this phase. Such visual cues ensured that participants had sufficient information to perform the tasks even in the absence of haptic feedback.

Upon arrival, participants were briefed on the study, and electromyography (EMG) and inertial measurement unit (IMU) sensors were placed. They then performed maximum voluntary contractions (MVC) to record baseline signals. Next, participants donned the VR headset and haptic armband to familiarize themselves with the virtual environment. The familiarization phase was flexible in length and lasted as long as each participant required to feel comfortable with the setup. After this, a randomly selected condition was explained, and during an acclimation phase, participants played the game under that condition until they demonstrated an understanding of the condition, which typically lasted approximately five minutes.

Once acclimated, the main data recording phase commenced, during which each participant performed two trials of the game under the given condition. After completing each condition, participants rested sufficiently to mitigate fatigue and, during this rest period, completed the NASA-TLx questionnaire to assess the workload experienced during that condition. At the end of the session, subjects also filled out a custom questionnaire to capture their overall perception of the system. Two experimenters were present during all sessions with healthy participants: one managed the VR and serious game software, while the other monitored and recorded data from the EMG and IMU sensors.

For participants with stroke, the primary goal was to assess feasibility rather than to collect quantitative data. Patients underwent a short (10-minute) acclimation phase, and conditions were presented in a randomized order. They had prior familiarity with virtual rehabilitation serious games and were briefed before the experiment. After each condition, they completed the NASA-TLx questionnaire and rested as needed before proceeding to the next condition. These sessions involved two experimenters (handling the VR and haptic systems) and a rehabilitation therapist, who supervised the session and also operated the serious game interface. At the end of the session, a semi-structured interview was conducted to gather participants’ perceptions of the device and its potential role in rehabilitation. They were asked whether they believed the device could help improve their motor skills in a rehabilitation setting, whether they would be likely to use it as part of a rehabilitation program, and whether they found the haptic armband comfortable to wear or experienced any irritation while using it.

### Performance metrics

#### Muscle activity

Surface electromyography (EMG) signals were recorded from key upper-limb muscles using wireless surface EMG sensors (Delsys Trigno, Delsys Inc., USA), following established electrode placement guidelines. Specifically, EMG was acquired from the shoulder muscles (anterior, posterior, and middle deltoids; infraspinatus; pectoralis clavicular), the elbow muscles (triceps long head; biceps short head; brachioradialis; pronator teres/supinator), and muscles involved in finger motion (extensor digitorum; flexor digitorum; abductor pollicis brevis). Signals were sampled at 2148.1 Hz, processed with a band-pass filter (30–450 Hz), rectified, and then low-pass filtered at 6 Hz using a zero-phase second-order Butterworth filter before being normalized to each subject’s maximum voluntary contraction (MVC). Finally, abnormal peaks and artifacts were removed using a moving median filter to prevent distortions in metric calculations and statistical analyses.

#### Kinematics

Kinematic data were collected using IMUs from the Xsens MVN Link system (Xsens Technologies B.V., Netherlands) at 240 Hz, with data processed using MVN Analyze software (version 2022.0.2, Xsens Technologies B.V., Netherlands). Segment-level joint kinematics were estimated using the built-in MVN biomechanical model^66^, which computes motion variables such as joint angles, segment velocities, and segment accelerations.

Through the vision-based hand tracking system embedded in the VR headset, the position, orientation, and pose of the hand were recorded and made available for processing in the developed VR application. The tracked hand pose (normalized by the system between 0 to 1 equal to an extended hand to a fully closed hand) and the pronation angle (computed as the angle between the lateral palm vector and the vertical axis in room coordinates) were used to compute the grasping and pronosupination error with respect to the given reference and in turn the haptic stimulation reference to send via local wi-fi network (UDP protocol) to the armband device.

#### Subjective analysis

To assess the perceived workload for each condition, participants completed the NASA Task Load Index (NASA-TLx) questionnaire, which evaluates mental demand (MD), physical demand (PD), temporal demand (TD), performance (PR), effort (EF), and frustration (FR). Furthermore, participants provided their overall perception of the system by completing a custom questionnaire that evaluated device comfort, potential irritation, and the interpretability, recognizability, reliance, precision, and responsiveness of both continuous and vibrotactile feedback (Table 1). Both questionnaires were scored based on the five-point Likert scale ranging from “strongly disagree” to “strongly agree.” It is important to note that, in our study, the NASA-TLX was adapted to a five-point Likert scale for each category, instead of the traditional 21-point scale. This was done to simplify the rating procedure within the immersive VR environment and to reduce the cognitive load for participants.

**Table 1:** Questionnaire Items and Corresponding Categories.

Question	Category
The armband felt comfortable to wear.	Comfort
I experienced discomfort or irritation due to pressure or vibrations.	Irritation
The continuous feedback (e.g., clenching or lateral stretch) was easy to interpret for task guidance.	Continuous Interpretability
The vibrotactile feedback was clear and helped me recognize the direction or extent of movement errors.	Vibrotactile Recognizability
I relied on the provided vibrotactile feedback to complete the tasks effectively.	Vibrotactile Reliance
I relied on the provided continuous feedback to complete the tasks effectively.	Continuous Reliance
Generally, I relied on the provided feedback to complete the tasks effectively.	Haptic Feedback Reliance
The feedback allowed me to guide my movements precisely.	Precision
The device’s feedback felt timely and responsive during the tasks.	Responsive Feedback

### Muscle synergy

Muscle synergies were computed for each participant and condition from the normalized EMG envelopes of all muscles using non-negative matrix factorization (NNMF)^67,68^. To reduce computation time, EMG data were downsampled to 500 Hz. Reconstruction quality was evaluated using the Variance Accounted For (VAF) metric^69^, which measures the proportion of variance captured. The optimal number of synergies was determined by two criteria: (1) when the slope of the VAF curve reached a predefined minimal change threshold, and (2) when the median VAF exceeded a target threshold. The final synergy count was chosen as the maximum value from these two criteria. We used a VAF threshold of 90 % and a slope threshold of 0.001 to determine the optimal number of synergies. Lastly, synergies were reordered across subjects using a similarity matrix based on a randomly selected reference subject.

### Data analysis and statistics

For all conditions, the EMG and IMU datasets were manually synchronized with the game phases extracted from the game data, and they were segmented based on the game phases. Within each game phase, we computed the root mean square (RMS) metric for both EMG signals (all muscles) and IMU data (position, velocity, and acceleration), averaged across repetitions. Additionally, Spectral Arc Length (SPARC)^70^ , and Range of Motion (ROM) metrics were calculated from the IMU signals. Pronosupination angles and grasping strength were extracted from the VR headset data as primary performance metrics. Finally, we computed a co-contraction index (CCI)^71^ for selected antagonistic muscle pairs (biceps–triceps, brachioradialis–triceps, flexor–extensor digitorum, anterior-posterior deltoids, pectoralis–posterior deltoid, and pronator teres–supinator). The statistical tests were computed using non-parametric tests, given the limited sample size. For each metric (i.e., EMG RMS, IMU RMS, SPARC, ROM, and CCI) and for each DoF, a preliminary inter-conditions test was performed using the Friedman statistical test, then pair-wise Wilcoxon signed rank tests were performed, in case of significance (i.e., *p* < 0.05) of the first test. Two three-condition comparisons were performed, focusing on the differences between types of feedback on the same motor action: for grasping motor action GV, GC, and NH conditions were compared whilst for pronosupination RV, RC and NH conditions were compared: in these cases Bonferroni correction for multiple tests was used, lowering the levels of significance threshold to $\alpha = 0.017$, $\alpha = 0.003$, and $\alpha = 0.0003$ for one, two, or three asterisks, considering three paired tests. Moreover, on muscle synergies, a Pearson correlation analysis was run comparing each condition with the NF condition, to check similarities of patterns: a paired Pearson test was run on each pair of vectors (i.e. synergies), reporting both the *R* and *p* values of the statistics: if significant, the test confirms the similarity of synergies.

## Data Availability

The final processed results used for statistical analysis and figure generation are available in the following repository: github.com/AliKMBonab/Haptic-Armband. Raw data and full synergy decomposition files are not publicly hosted due to size and privacy considerations, but are available from the corresponding author upon reasonable request.

## References

[1] Hendricks, H. T., Van Limbeek, J., Geurts, A. C. & Zwarts, M. J. Motor recovery after stroke: A systematic review of the literature. *Arch. Phys. Med. Rehabil.* **83**, 1629–1637 (2002). 12422337 10.1053/apmr.2002.35473

[2] Langhorne, P., Coupar, F. & Pollock, A. Motor recovery after stroke: A systematic review. *Lancet Neurol.* **8**, 741–754 (2009). 19608100 10.1016/S1474-4422(09)70150-4

[3] Faria-Fortini, I., Michaelsen, S. M., Cassiano, J. G. & Teixeira-Salmela, L. F. Upper extremity function in stroke subjects: Relationships between the international classification of functioning, disability, and health domains. *J. Hand Ther.* **24**, 257–265 (2011). 21420279 10.1016/j.jht.2011.01.002

[4] Carod-Artal, F. J. & Egido, J. A. Quality of life after stroke: The importance of a good recovery. *Cerebrovasc. Dis.* **27**, 204–214 (2009). 19342853 10.1159/000200461

[5] Lohse, K. R., Lang, C. E. & Boyd, L. A. Is more better? Using metadata to explore dose-response relationships in stroke rehabilitation. *Stroke* **45**, 2053–2058 (2014). 24867924 10.1161/STROKEAHA.114.004695 PMC4071164

[6] Winstein, C. J. et al. Guidelines for adult stroke rehabilitation and recovery: A guideline for healthcare professionals from the American heart association/american stroke association. *Stroke* **47**, e98–e169 (2016). 27145936 10.1161/STR.0000000000000098

[7] Gadidi, V., Katz-Leurer, M., Carmeli, E. & Bornstein, N. M. Long-term outcome poststroke: Predictors of activity limitation and participation restriction. *Arch. Phys. Med. Rehabil.* **92**, 1802–1808 (2011). 22032214 10.1016/j.apmr.2011.06.014

[8] Kim, W.-S. et al. Clinical application of virtual reality for upper limb motor rehabilitation in stroke: Review of technologies and clinical evidence. *J. Clin. Med.* **9**, 3369 (2020). 33096678 10.3390/jcm9103369 PMC7590210

[9] Chen, Y. et al. Home-based technologies for stroke rehabilitation: A systematic review. *Int. J. Med. Informatics* **123**, 11–22 (2019). 10.1016/j.ijmedinf.2018.12.001 PMC6814146 30654899

[10] Ceradini, M., Losanno, E., Micera, S., Bandini, A. & Orlandi, S. Immersive vr for upper-extremity rehabilitation in patients with neurological disorders: A scoping review. *J. Neuroeng. Rehabil.* **21**, 75 (2024). 38734690 10.1186/s12984-024-01367-0 PMC11088157

[11] Levin, M. F., Weiss, P. L. & Keshner, E. A. Emergence of virtual reality as a tool for upper limb rehabilitation: Incorporation of motor control and motor learning principles. *Phys. Ther.* **95**, 415–425 (2015). 25212522 10.2522/ptj.20130579 PMC4348716

[12] Elor, A., Teodorescu, M. & Kurniawan, S. Project star catcher: A novel immersive virtual reality experience for upper limb rehabilitation. *ACM Trans. Access. Comput. (TACCESS)* **11**, 1–25 (2018).

[13] Burke, J. W. et al. Optimising engagement for stroke rehabilitation using serious games. *Vis. Comput.* **25**, 1085–1099 (2009).

[14] Wang, L., Chen, J.-L., Wong, A. M., Liang, K.-C. & Tseng, K. C. Game-based virtual reality system for upper limb rehabilitation after stroke in a clinical environment: Systematic review and meta-analysis. *Games Health J.* **11**, 277–297 (2022). 36252097 10.1089/g4h.2022.0086

[15] Bortone, I. et al. Immersive virtual environments and wearable haptic devices in rehabilitation of children with neuromotor impairments: A single-blind randomized controlled crossover pilot study. *J. Neuroeng. Rehabil.* **17**, 1–14 (2020). 33115487 10.1186/s12984-020-00771-6 PMC7594483

[16] Adlakha, S., Chhabra, D. & Shukla, P. Effectiveness of gamification for the rehabilitation of neurodegenerative disorders. *Chaos, Solitons Fractals* **140**, 110192 (2020).

[17] Elor, A. & Kurniawan, S. The ultimate display for physical rehabilitation: A bridging review on immersive virtual reality. *Front. Virtual Real.* **1**, 585993 (2020).

[18] Heinrich, C., Morkisch, N., Langlotz, T., Regenbrecht, H. & Dohle, C. Feasibility and psychophysical effects of immersive virtual reality-based mirror therapy. *J. Neuroeng. Rehabil.* **19**, 107 (2022). 36207720 10.1186/s12984-022-01086-4 PMC9540740

[19] Park, W., Kim, J. & Kim, M. Efficacy of virtual reality therapy in ideomotor apraxia rehabilitation: A case report. *Medicine* **100**, e26657 (2021). 34260571 10.1097/MD.0000000000026657 PMC8284726

[20] Erhardsson, M., Alt Murphy, M. & Sunnerhagen, K. S. Commercial head-mounted display virtual reality for upper extremity rehabilitation in chronic stroke: A single-case design study. *J. Neuroeng. Rehabil.* **17**, 1–14 (2020). 33228710 10.1186/s12984-020-00788-x PMC7686731

[21] Vourvopoulos, A. et al. Efficacy and brain imaging correlates of an immersive motor imagery bci-driven vr system for upper limb motor rehabilitation: A clinical case report. *Front. Hum. Neurosci.* **13**, 244 (2019). 31354460 10.3389/fnhum.2019.00244 PMC6637378

[22] Kamm, C. P., Blättler, R., Kueng, R. & Vanbellingen, T. Feasibility and usability of a new home-based immersive virtual reality headset-based dexterity training in multiple sclerosis. *Multiple Sclerosis Related Disord.* **71**, 104525 (2023). 10.1016/j.msard.2023.104525 36738693

[23] Chau, B. et al. Immersive virtual reality therapy with myoelectric control for treatment-resistant phantom limb pain: Case report. *Innov. Clin. Neurosci.* **14**, 3 (2017). 29616149 PMC5880370

[24] Osumi, M. et al. Restoring movement representation and alleviating phantom limb pain through short-term neurorehabilitation with a virtual reality system. *Eur. J. Pain* **21**, 140–147 (2017). 27378656 10.1002/ejp.910

[25] Booth, A. T., Buizer, A. I., Harlaar, J., Steenbrink, F. & van der Krogt, M. M. Immediate effects of immersive biofeedback on gait in children with cerebral palsy. *Arch. Phys. Med. Rehabil.* **100**, 598–605 (2019). 30447196 10.1016/j.apmr.2018.10.013

[26] Levin, M. F., Michaelsen, S. M., Cirstea, C. M. & Roby-Brami, A. Use of the trunk for reaching targets placed within and beyond the reach in adult hemiparesis. *Exp. Brain Res.* **143**, 171–180 (2002). 11880893 10.1007/s00221-001-0976-6

[27] Latash, M. L. & Anson, J. G. What are “normal movements’’ in atypical populations?. *Behav. Brain Sci.* **19**, 55–68 (1996).

[28] Da Gama, A., Chaves, T., Figueiredo, L. & Teichrieb, V. Guidance and movement correction based on therapeutics movements for motor rehabilitation support systems. In *2012 14th symposium on virtual and augmented reality* 191–200 (IEEE, 2012).

[29] Sanford, S., Collins, B., Liu, M., Dewil, S. & Nataraj, R. Investigating features in augmented visual feedback for virtual reality rehabilitation of upper-extremity function through isometric muscle control. *Front. Virtual Reality* **3**, 943693 (2022).

[30] Xiao, B. et al. Design of a virtual reality rehabilitation system for upper limbs that inhibits compensatory movement. *Med. Novel Technol. Dev.* **13**, 100110 (2022).

[31] Frisoli, A. & Leonardis, D. Wearable haptics for virtual reality and beyond. *Nat. Rev. Electr. Eng.* **1**, 666–679 (2024).

[32] Pacchierotti, C. et al. Wearable haptic systems for the fingertip and the hand: Taxonomy, review, and perspectives. *IEEE Trans. Haptics* **10**, 580–600 (2017). 28500008 10.1109/TOH.2017.2689006

[33] Jakob, I. et al. Robotic and sensor technology for upper limb rehabilitation. *PM&R* **10**, S189–S197 (2018). 30269805 10.1016/j.pmrj.2018.07.011

[34] Abbate, G., Giusti, A., Randazzo, L. & Paolillo, A. A mirror therapy system using virtual reality and an actuated exoskeleton for the recovery of hand motor impairments: a study of acceptability, usability, and embodiment. *Sci. Rep.* **13**, 22881 (2023). 38129489 10.1038/s41598-023-49571-7 PMC10739894

[35] Lugo-Villeda, L. I. et al. Haptic guidance of light-exoskeleton for arm-rehabilitation tasks. In *RO-MAN 2009-The 18th IEEE International Symposium on Robot and Human Interactive Communication*, 903–908 (IEEE, 2009).

[36] Liu, X. et al. Design of virtual guiding tasks with haptic feedback for assessing the wrist motor function of patients with upper motor neuron lesions. *IEEE Trans. Neural Syst. Rehabil. Eng.* **27**, 984–994 (2019). 30969927 10.1109/TNSRE.2019.2909287

[37] Islam, M. S. & Lim, S. Vibrotactile feedback in virtual motor learning: A systematic review. *Appl. Ergon.* **101**, 103694 (2022). 35086007 10.1016/j.apergo.2022.103694

[38] Kim, K., Yang, H., Lee, J. & Lee, W. G. Metaverse wearables for immersive digital healthcare: A review. *Adv. Sci.* **10**, 2303234 (2023). 10.1002/advs.202303234 PMC10625124 37740417

[39] Eguchi, R., Vacek, D., Godzinski, C. & Okamura, A. M. Between-tactor display using dynamic tactile stimuli for directional cueing in vibrating environments. *IEEE Trans. Haptics* (2023). 10.1109/TOH.2023.3304953 37578911

[40] Scheggi, S., Talarico, A. & Prattichizzo, D. A remote guidance system for blind and visually impaired people via vibrotactile haptic feedback. In *22nd Mediterranean Conference on Control and Automation* 20–23 (IEEE, 2014).

[41] Barontini, F., Catalano, M. G., Pallottino, L., Leporini, B. & Bianchi, M. Integrating wearable haptics and obstacle avoidance for the visually impaired in indoor navigation: A user-centered approach. *IEEE Trans. Haptics* **14**, 109–122 (2020). 10.1109/TOH.2020.2996748 32746372

[42] Fani, S., Ciotti, S. & Bianchi, M. Multi-cue haptic guidance through wearables for enhancing human ergonomics. *IEEE Trans. Haptics* **15**, 115–120 (2021). 10.1109/TOH.2021.3137899 34941521

[43] Kim, W., Garate, V. R., Gandarias, J. M., Lorenzini, M. & Ajoudani, A. A directional vibrotactile feedback interface for ergonomic postural adjustment. *IEEE Trans. Haptics* **15**, 200–211 (2021). 10.1109/TOH.2021.3112795 34529575

[44] Rätz, R., Ratschat, A. L., Cividanes-Garcia, N., Ribbers, G. M. & Marchal-Crespo, L. Designing for usability: Development and evaluation of a portable minimally-actuated haptic hand and forearm trainer for unsupervised stroke rehabilitation. *Front. Neurorobot.* **18**, 1351700 (2024). 38638360 10.3389/fnbot.2024.1351700 PMC11024237

[45] Frisoli, A. et al. A randomized clinical control study on the efficacy of three-dimensional upper limb robotic exoskeleton training in chronic stroke. *J. Neuroeng. Rehabil.* **19**, 14 (2022). 35120546 10.1186/s12984-022-00991-y PMC8817500

[46] Osu, R. et al. Optimal impedance control for task achievement in the presence of signal-dependent noise. *J. Neurophysiol.* **92**, 1199–1215 (2004). 15056685 10.1152/jn.00519.2003

[47] Gribble, P. L., Mullin, L. I., Cothros, N. & Mattar, A. Role of cocontraction in arm movement accuracy. *J. Neurophysiol.* **89**, 2396–2405 (2003). 12611935 10.1152/jn.01020.2002

[48] Palastanga, N., Field, D. & Soames, R. *Anatomy and human movement: structure and function*, vol. 20056 (Elsevier Health Sciences, 2006).

[49] Kleiber, T., Kunz, L. & Disselhorst-Klug, C. Muscular coordination of biceps brachii and brachioradialis in elbow flexion with respect to hand position. *Front. Physiol.* **6**, 215 (2015). 26300781 10.3389/fphys.2015.00215 PMC4526813

[50] Escamilla, R. F., Yamashiro, K., Paulos, L. & Andrews, J. R. Shoulder muscle activity and function in common shoulder rehabilitation exercises. *Sports Med.* **39**, 663–685 (2009). 19769415 10.2165/00007256-200939080-00004

[51] Wickens, C. D. Multiple resources and mental workload. *Hum. Factors* **50**, 449–455 (2008). 18689052 10.1518/001872008X288394

[52] Marucci, M. et al. The impact of multisensory integration and perceptual load in virtual reality settings on performance, workload and presence. *Sci. Rep.* **11**, 4831 (2021). 33649348 10.1038/s41598-021-84196-8 PMC7921449

[53] Wang, G., Wang, H.-H. & Ren, G. Visual and haptic guidance for enhancing target search performance in dual-task settings. *Appl. Sci.* **14**, 4650 (2024).

[54] Camponogara, I. & Volcic, R. Visual uncertainty unveils the distinct role of haptic cues in multisensory grasping. *Eneuro* **9**, (2022). 10.1523/ENEURO.0079-22.2022 PMC9215692 35641223

[55] Spaccavento, S. et al. Attention deficits in stroke patients: The role of lesion characteristics, time from stroke, and concomitant neuropsychological deficits. *Behav. Neurol.* **2019**, 7835710 (2019). 31263512 10.1155/2019/7835710 PMC6556322

[56] Hepworth, L. R. et al. Post-stroke visual impairment: A systematic literature review of types and recovery of visual conditions. *Ophthalmol. Res.: Int. J.* **5**, 1–43 (2016).

[57] Held, J. P., Klaassen, B., Van Beijnum, B.-J.F., Luft, A. R. & Veltink, P. H. Usability evaluation of a vibrotactile feedback system in stroke subjects. *Front. Bioeng. Biotechnol.* **4**, 98 (2017). 28180128 10.3389/fbioe.2016.00098 PMC5263126

[58] Du, Q., Luo, J., Cheng, Q., Wang, Y. & Guo, S. Vibrotactile enhancement in hand rehabilitation has a reinforcing effect on sensorimotor brain activities. *Front. Neurosci.* **16**, 935827 (2022). 36267238 10.3389/fnins.2022.935827 PMC9577243

[59] Seim, C. E., Wolf, S. L. & Starner, T. E. Wearable vibrotactile stimulation for upper extremity rehabilitation in chronic stroke: Clinical feasibility trial using the vts glove. *J. Neuroeng. Rehabil.* **18**, 1–11 (2021). 33485371 10.1186/s12984-021-00813-7 PMC7824932

[60] Devigne, L. et al. Power wheelchair navigation assistance using wearable vibrotactile haptics. *IEEE Trans. Haptics* **13**, 52–58 (2020). 31905149 10.1109/TOH.2019.2963831

[61] Aggravi, M., Salvietti, G. & Prattichizzo, D. Haptic assistive bracelets for blind skier guidance. In *Proceedings of the 7th Augmented Human International Conference 2016* 1–4 (2016).

[62] Bark, K. et al. Effects of vibrotactile feedback on human learning of arm motions. *IEEE Trans. Neural Syst. Rehabil. Eng.* **23**, 51–63 (2014). 25486644 10.1109/TNSRE.2014.2327229 PMC4623827

[63] Basalp, E., Wolf, P. & Marchal-Crespo, L. Haptic training: Which types facilitate (re) learning of which motor task and for whom? Answers by a review. *IEEE Trans. Haptics* **14**, 722–739 (2021). 34388095 10.1109/TOH.2021.3104518

[64] Abdlkarim, D. et al. A methodological framework to assess the accuracy of virtual reality hand-tracking systems: A case study with the meta quest 2. *Behav. Res. Methods* **56**, 1052–1063 (2024). 36781700 10.3758/s13428-022-02051-8 PMC10830632

[65] Barontini, F. et al. The cuff, clenching upper-limb force feedback wearable device: Design, characterization and validation. *IEEE Trans. Haptics* (2024). 10.1109/TOH.2024.3375010 38478436

[66] Roetenberg, D., Luinge, H., Slycke, P. et al. Xsens mvn: Full 6dof human motion tracking using miniature inertial sensors. *Xsens Motion Technologies BV, Tech. Rep*vol. 1, pp. 1–7 (2009).

[67] Lee, D. D. & Seung, H. S. Learning the parts of objects by non-negative matrix factorization. *Nature* **401**, 788–791 (1999). 10548103 10.1038/44565

[68] Lee, D. & Seung, H. S. Algorithms for non-negative matrix factorization. *Adv. Neural Inf. Process. Syst.* **13**, (2000).

[69] Torres-Oviedo, G., Macpherson, J. M. & Ting, L. H. Muscle synergy organization is robust across a variety of postural perturbations. *J. Neurophysiol.* **96**, 1530–1546 (2006). 16775203 10.1152/jn.00810.2005

[70] Balasubramanian, S., Melendez-Calderon, A., Roby-Brami, A. & Burdet, E. On the analysis of movement smoothness. *J. Neuroeng. Rehabil.* **12**, 1–11 (2015). 26651329 10.1186/s12984-015-0090-9 PMC4674971

[71] Steele, K. M., Jackson, R. W., Shuman, B. R. & Collins, S. H. Muscle recruitment and coordination with an ankle exoskeleton. *J. Biomech.* **59**, 50–58 (2017). 28623037 10.1016/j.jbiomech.2017.05.010 PMC5644499

